# Functional Marker Assisted Improvement of Stable Cytoplasmic Male Sterile Lines of Rice for Bacterial Blight Resistance

**DOI:** 10.3389/fpls.2017.01131

**Published:** 2017-06-29

**Authors:** Jegadeesan Ramalingam, Palanisamy Savitha, Ganesh Alagarasan, Ramasamy Saraswathi, Ranganathan Chandrababu

**Affiliations:** ^1^Department of Plant Molecular Biology and Bioinformatics, Centre for Plant Molecular Biology and Biotechnology, Tamil Nadu Agricultural UniversityCoimbatore, India; ^2^Department of Rice, Center for Plant Breeding and Genetics, Tamil Nadu Agricultural UniversityCoimbatore, India

**Keywords:** bacterial blight resistance, functional markers, hybrid rice, cytoplasmic male sterility, marker-assisted backcross breeding, foreground selection, background selection, fertility restoration

## Abstract

Bacterial blight (BB), caused by *Xanthomonas oryzae pv*.oryzae is one among the major diseases in rice, which in severe condition cause losses up to 60% in total yield. Marker assisted pyramiding of three broad spectrum BB resistance genes (*xa5, xa13*, and *Xa21*) in prominent rice varieties is the most economical and effective strategy for the management of the BB disease. We report here the pyramiding of three genes (*xa5, xa13*, and *Xa21*) in maintainer lines (CO 2B, CO 23B, and CO 24B) of three promising wild abortive cytoplasmic male sterile lines (CO 2A, CO 23A, and CO 24A) through functional markers assisted back cross breeding. IRBB60 with *xa5, xa13*, and *Xa21* genes is used as a donor parent. BC_2_F_1_ and BC_2_F_2_ generations from a cross of CO 2B, CO 23B, and CO 24B with IRBB60 were evaluated for bacterial blight and non-fertility restoration. In BC_2_F_1_, plants with all three resistance genes (*xa5, xa13*, and *Xa21*) and high parent genome recovery was identified. In BC_2_F_2_, plants with all resistance genes and without fertility restorer (*Rf3* and *Rf4*) were selected. Based on agronomic traits, BB resistance and maintenance of sterility, two plants each in CO 2B × IRBB60, CO 24B × IRBB60 and one plant in CO 23B × IRBB60 combinations were identified. The identified lines were crossed with respective male sterile lines for conversion of improved B line into CMS line through back-crossing, in addition to selfing. The plants with high recurrent genome and phenotypically similar to parental lines and sterile are being used for the hybrid rice development program. Currently, using these lines (improved CMS line), test crosses were made to develop new rice hybrids. Hybrids combinations *viz.*, CO 23A × AD08009R and CO 24A × IET20898R were found to be stable at different locations with high yield. The R line used in this study has been introgressed with *xa5, xa13*, and *Xa21* genes in a separate breeding program. These new hybrids with resistance against bacterial blight will increase the crop production at BB environment.

## Introduction

Rice (*Oryza sativa* L.) is one of the staple food crops and grown in a wide range of climatic conditions. Blight is an important bacterial disease in rice (Gnanamanickam et al., 1999), hence developing a BB resistant rice variety can give real time solution to the farmers. To develop resistant varieties, functional markers have a selective advantage over indirect selection using molecular markers (Andersen and Lübberstedt, 2003; Varshney et al., 2005; Iyer-Pascuzzi and Mccouch, 2007; Ingvardsen et al., 2008). Due to the lack of diverse parental lines with desirable traits and multiple resistances, hybrid rice production in India depends mostly on parental lines developed in China and IRRI. These outsourced lines are not well adapted to the Indian climate, resulting in poor grain quality. Therefore, we have decided to improve native CMS and maintainer line for biotic and abiotic stresses with higher production and productivity focusing on hybrid rice development. Currently, 10 CMS lines of native origin were developed at our University using Wild abortive (WA) cytoplasmic source. Quite a good number of stable hybrids were developed with these native CMS lines. Two hybrids, CORH 3 and CORH 4 were released recently from our center. CORH 4 is very popular among the Southern part of India. Low adaptability of earlier released hybrids is due to poor seed setting and grain quality. The hybrids developed using CO 23A overcomes the problem of low adaptability and poor grain setting shown by the earlier released hybrids, but the hybrids and their parental lines are more prone to bacterial blight disease.

Gene pyramid work has been successfully employed in several crops for agronomically important traits. Basavaraj et al. (2010) had successfully introgressed two bacterial blight resistance genes (*xa13* and *Xa21*) into parental lines Pusa 6B and PRR 78 of Pusa RH10 hybrid. The elite restorer line of hybrid rice, Minghui 63, which become more susceptible to bacterial blight was improved for resistance through introgression of Xa21 gene within 3 generations of back-crossing (Chen and Ronald, 1999). A novel bacterial blight resistance gene *Xa23*, identified in wild rice species *Oryza rufipogaon* was reported to confer resistance to 20 races of bacterial blight from China, India and Philippines right through all the growth stages of rice (Zhang et al., 2002; Zhang, 2009). The restorer lines Minghui 63, Y1671 and YR293 improved with the introgression of Xa23 gene revealed to have a wide range of resistance to bacterial blight (Zhou et al., 2011). Recently, more reliable Marker assisted selection is gaining significance in the field of disease resistance breeding for hybrid rice production. The elite restorer line RPHR 1005 was improved for BB and blast resistance with the aid of gene specific markers (Kumar et al., 2016).

Promising BB resistance genes viz., *Xa21, xa13, Xa4*, and *xa5* with substantial resistance was introgressed into the background of restorer (KMR 3 and PRR 78) and maintainer lines (IR 58025B and Pusa 6B) through MAS (Shanti et al., 2010). Chen et al. (2000) successfully incorporated BB resistance genes by MAS. Successful enhancement of resistance to bacterial blight in an elite restorer line Hau 1035 was accomplished by pyramiding *Xa23, Xa22, Xa21*, and *Xa7* resistance genes by means of MAS (Huang et al., 2012). Pusa RH 1000, super fine rice hybrid and its parental lines- Pusa6B and PRR78 were improved for resistance to BB (*xa13* and *Xa21*) along with agronomic, grain and cooking quality traits through MAS. Similarly, the maintainer line (DRR 17B) and restorer line (RPHR-1005) were improved for BB resistance (Xa21) and blast resistance (Pi54) through MAS (Balachiranjeevi et al., 2015; Kumar et al., 2016). Based on the earlier reports and our hypothesis, we selected two BB resistant recessive genes (*xa5* and *xa13*) and one dominant gene (*Xa21*). Therefore, incorporation of three BB resistant genes (*xa5, xa13*, and *Xa21*) combination was taken up in the WA-CMS maintainer lines (CO 2B, CO 23B, and CO 24B) through back-crossing without fertility restorer genes (*Rf3* and *Rf4*) of WA-CMS system using functional markers.

## Materials and methods

### Development of plant population

**Figure 1:**
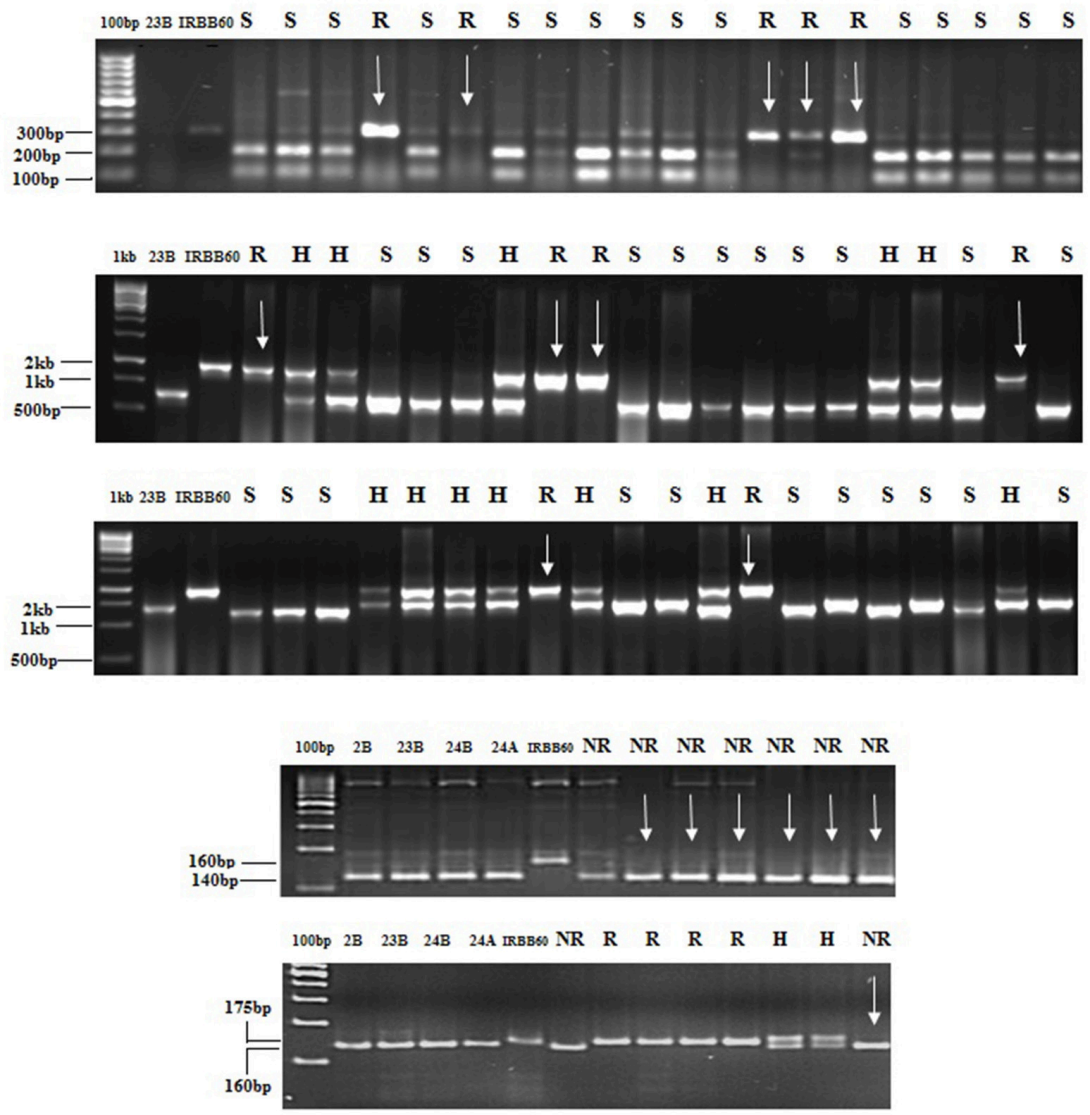
Foreground selection in progenies. PCR amplification of BC_2_F_2_ plants of CO 23B × IRBB 60 cross combination (1) xa5_1F, xa5_1R restricted with the enzyme BsrI to detect polymorphism (2) with *xa13* (3) with Xa21 (4) DRRM-RF3-10 maker for *Rf*_3_ genes (5) RM 6100 marker for *Rf*_4_ In figure, R, H, S indicates Resistant, heterozygote and susceptible respectively. CO2B, CO 23B, CO 24 B, and CO 24A were susceptible genotypes and IRBB60 was resistant source.

**Figure 2:**
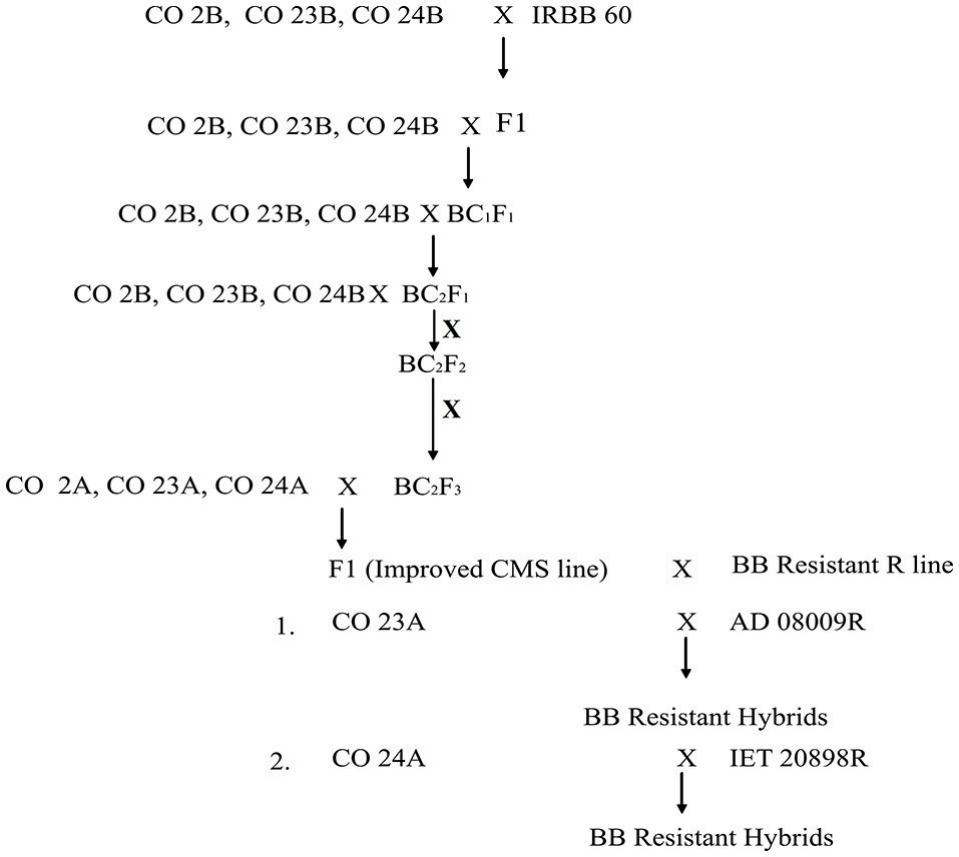
Schematic representation of the introgression of xa5, xa13, and Xa21 into the maintainer and CMS line.

The experimental material consisted of CO 2A, CO 23A, CO 24A of WA cytoplasmic source and its maintainer, CO 2B, CO 23B, and CO 24B, as recurrent parents. IRBB 60 (BB donor line), IR 24 (susceptible check) and identified BB resistant restorer lines (AD 08009, AD 09525, IET 19863, IET 20885, IET 20897, and IET 20898) of WA cytoplasmic source were used as the genetic material for the present study. Pure seeds of rice lines were obtained from the Paddy Breeding Station, Centre for Plant Breeding and Genetics (CPBG), Coimbatore. The plant population was developed and maintained at the above mentioned center. Genomic DNA was extracted using modified CTAB method from freshly collected leaves (45 DAS) and its purity was assessed by Nanodrop™ 1,000 Spectrophotometer. PCR amplification of genes (*xa5, xa13*, and *Xa21*) for all the parents, F_1_, BC_1_F_1_,BC_2_F_1_, BC_2_F_2_, and BC_2_F_3_ generations from the cross of CO 2B, CO 23B, and CO 24B with IRBB60 respectively, and bacterial blight screening was carried out as described in our earlier study (Perumalsamy et al., 2010). PCR details have been provided in Datasheet S1. The amplified products were resolved in 2.5% agarose gel (Figure 1). The crossing layout of hybrid development is provided in Figure 2. Pollens were collected in randomly selected F_2_ plants from a cross of A × R line.

Pollen grains were squeezed out and placed on a glass slide for staining with (I- KI). Deep stained and round sized pollens were considered as fertile. Spikelet fertility was checked from the panicles of selected plants. Number of filled seeds to the number of spikelet in a panicle were taken into account to predict spikelet fertility.

### Functional marker analysis

Functional marker for *xa5* gene (Iyer-Pascuzzi and Mccouch, 2007), *xa13* gene (Chu et al., 2006), and *Xa21* gene (Perumalsamy et al., 2010) were used. For fertility restoration, markers linked to *Rf3* and *Rf4* were used. Three SSR markers, comprising one RM marker - RM 6100 (160 bp in non-restorer line and 175 bp for restorer line), two candidate gene markers DRCG-*RF4*-14 (885 bp in non-restorer line and 845 bp for restorer line) and DRCG-*RF4*-8(1096 bp in non-restorer line and 760 bp for restorer line) for *Rf4* locus and two SSR markers DRRM-RF3-5 (140 bp in non-restorer line and 160 bp for restorer line) and DRRM-RF3-10 (140 bp in non-restorer line and 150 bp for restorer line) for *Rf3* reported by Suresh et al. (2012) were used in BC_2_F_2_ and BC_2_F_3_ generations. The details of markers sequences were given in Table 1. To examine parental polymorphism between donor and elite parents, microsatellite markers covering the whole rice genome were utilized. The primer information and chromosomal position were retrieved from the Gramene database (www.gramene.org).

**Table 1:** Details of markers used for bacterial blight resistance and fertility restoration.

**S.No**	**Marker**	**Primer Sequences 5′ → 3′**	**Chromosomal location**	**Authors**
**BACTERIAL BLIGHT RESISTANCE**				
1.	xa 5-1F	F-ACGCTCGACGAGATGGTCTC	5	Iyer-Pascuzzi and Mccouch, 2007
	xa 5-1R	R- ATCACAAGCGCATATATGAG		
2.	xa 13	F- AGCTCCAGCTCCAAATG	8	Chu et al., 2006
		R- GGCCATGGCTCAGTGTTTAT		
3.	Xa 21	F- ATAGCTAGTTCATAGAGG	7	Perumalsamy et al., 2010
		R- ACATCCGTCACTCTTGCCA		
***Rf*_*3*_ LOCUS**				
4.	DRRM-RF3-5	F- GATGGCACAGCTTCAGAACA	1	Suresh et al., 2012
		R-CTAATTCTGGGCGAGCAAAG		
5.	DRRM-RF3-10	F- TCACCTCTTCCTGCTTCGAC	1	Suresh et al., 2012
		R- CTCCACCAGTGCAGGTTTT		
***Rf*_*4*_ LOCUS**				
6.	DRCG-RF4-14	F- GCAATGCTTGTATTCAGCAAA	10	Suresh et al., 2012
		R-TCCAGCTGTAAATCCGTCAA		
7.	DRCG-RF4-8	F- TTGCAACGCAAGGGTAATTT	10	Suresh et al., 2012
		R- TCACTGCGCATCTTTTTGAG		
8.	RM 6100	F- TCCTCTACCAGTACCGCACC	10	Sheeba et al., 2009
		R- GCTGGATCACAGATCATTGC		

The plants were scored as B (maintainer indicates allele), H (heterozygous state), and NR (non-fertility restorer genes). For fertility restoration genes (*Rf3, Rf4*), the amplified PCR product was separated in 3 per cent agarose gel.

## Results

### Introgression of BB resistance genes in maintainer line and CMS line

Functional markers were used to incorporate both BB genes (*xa5, xa13*, and *Xa21*) and the two major *Rf* genes (*Rf3* and *Rf4*) governing fertility restoration of cytoplasmic-geneic male sterility (CMS) in our targeted rice varieties. During the breeding procedure, foreground selection was practiced from the F_1_ generation to BC_2_F_2_ generation. At each stage, the plants having resistance alleles of the three target genes were selected and only progenies having resistance alleles of the three target genes were advanced to the next generation. A total of 20 (CO 2B), 16 (CO 23B), and 10 (CO 24B) plants were identified to be true F_1_s and back crossed to respective recurrent parents.

**Figure 3:**
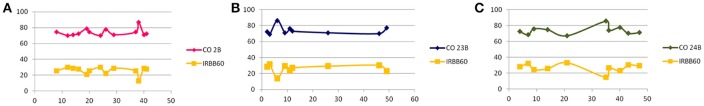
Graphical representation of background analysis. Y- axis represents the percentage of recurrent parent genome recovery using polymorphic SSR markers distributed all over the 12 chromosomes (RPG_SSR_) in BC_1_F_1_ generation **(A)** CO 2B × IRBB60, **(B)** CO 23B × IRBB60, and **(C)** CO 24B × IRBB60. While, X- axis indicates the number of agro-morphologically selected plants from foreground analysis subjected to RPG_SSR_ analysis carrying *xa5, xa13*, and *Xa21* gene combination.

In BC_1_F_1_, foreground selection with functional markers of target genes resulted in identification of heterozygous plants in all three crosses. A total of 13/60 (CO 2B), 2/58 (CO 23B), and 2/65 (CO 24 B) plants were found to be triple heterozygous for the target “R” gene functional markers. Background analysis was carried out with polymorphic SSR markers to assess the recovery of recurrent genome contribution in identified triple heterozyogus plants in all three combinations. Resultant triple gene heterozygous (*Xa21Xa21, Xa13xa13*, and *Xa5xa5*) plants with maximum recovery of the recurrent genome in all three crosses, CO 2B (74.2%), CO 23B (73.8%), and CO 24B (73.5%) were further back crossed with their respective recurrent parents (Figure 3).

In BC_2_F_1_, a total of 12/96 (CO 2B), 9/101 (CO 23B), and 9/89 (CO 24B) plants were found to be triple heterozygous for *xa5, xa13*, and *Xa 21* genes. The heterozygous plants were subjected to back-ground selection using polymorphic SSR markers. Single triple heterozygous plant with maximum recovery of recurrent parent in CO 2B (87.1%), CO 23B (86.2%), and CO 24B (85.2%) were selfed and forwarded to F_2_ generation (Figure 3). In BC_2_F_2_ generation, a total of 190 (CO 2B), 160 (CO 23B), and 120 (CO 24 B) were subjected to PCR analysis using FMs (Figure 1). The segregation pattern for bacterial blight resistance in BC_2_F_2_ was tested using chi square (χ^2^) analysis. The segregation of all the three resistance genes was insignificant as the calculated χ^2^ value is lower than the table values at 5% (5.99) and 1% (9.21) levels (Table 2). Plants with single, double, and triple gene combinations with BB resistance were identified. In CO 2B × IRBB 60, forty one plant with *Xa21* gene, 52 plants with *xa13* and 43 plants with the recessive gene *xa5* in homozygous condition were identified. Fifteen plants homozygous for two genes *xa5* and *xa13*, 10 for *xa5* and *Xa21* and 10 plants for *xa13* and *Xa21* were identified. Twelve plants possessing all the three genes, *xa5, xa13*, and *Xa21* in homozygous conditions were identified. Similarly, in CO 23B x IRBB60 and CO 24 B × IRBB 60 plants with different resistance genes combinations were obtained. Plants with triple homozygous for target genes in all three crosses, 12/190 (CO 2B), 9/160 (CO 23B), and 9/120 (CO 24 B) were found.

**Table 2:** Segregating ratio of the marker genotypes in BC_2_F_2_ generation for BB.

**S.No**	**Markers**	**2B × IRBB60**					**23B × IRBB60**					**24B × IRBB60**				
		**Observed frequency**					**Observed frequency**					**Observed frequency**				
		**RR**	**Rr**	**Rr**	**Total**	**χ^2^ (1:2:1)**	**RR**	**Rr**	**rr**	**Total**	**χ^2^ (1:2:1)**	**RR**	**Rr**	**rr**	**Total**	**χ^2^ (1:2:1)**
1.	Xa21 (F&R)	41	104	45	190	1.873	32	77	51	160	4.237	28	59	33	120	0.449
2.	xa13 (F&R)	52	83	55	190	3.126	37	72	51	160	4.437	39	54	27	120	3.600
3.	xa5 (2F&3R)	43	97	50	190	0.599	29	78	53	160	4.950	32	54	34	120	1.266

*RR, rr,Homozygotes; Rr, Heterozygote*.

### Bacterial blight screening

**Figure 4:**
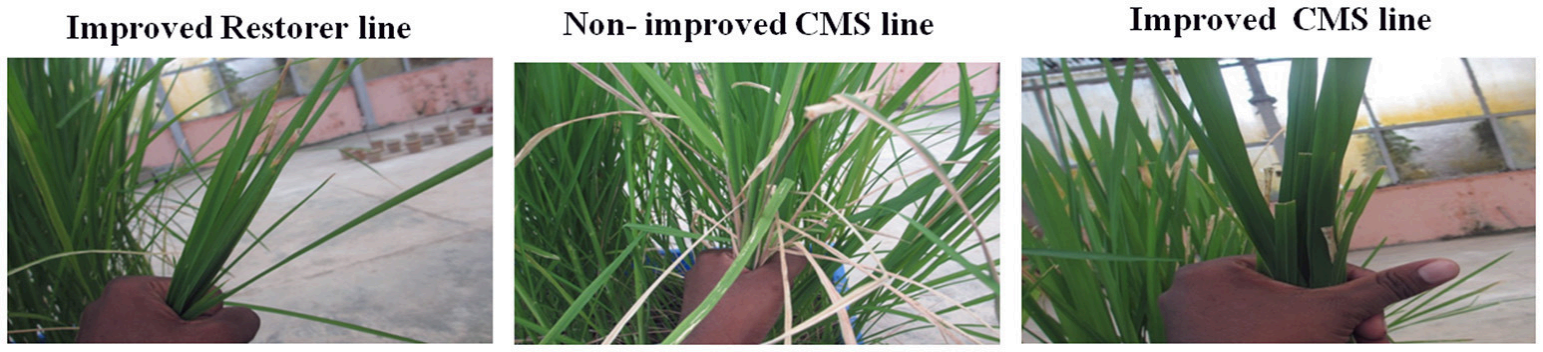
Differential reactions of *Xoo* isolates on rice leaves at 14 dpi upon artificial clip inoculation.

The selected backcross-derived 30 homozygous lines from three crosses along with recurrent parents, donor line and susceptible check were evaluated for their reactions to five predominantly available races of bacterial blight pathogen under green house condition. A representative picture is given as Figure 4. The mean lesion lengths of plants with three resistance genes in BC2F3 population of three crosses were given in Table 3. As expected, all the 30 homozygous plants with three resistance genes (i.e., plants with genotype of *xa5xa5, xa13xa13*, and *Xa21Xa21)* showed higher levels of resistance with a mean lesion length of less than 5.0 cm for all the races with few exceptions. The susceptible check, IR 24 and recurrent parents, CO 2B, CO 23 B, and CO 24 B showed more than 15.0 cm of lesion length and categorized as highly susceptible to bacterial blight pathogen. The donor parent, IRBB 60 showed high level of resistance with mean lesion length of 4.53 cm.

**Table 3:** Mean lesion length of *Xoo* pathotypes on pyramided lines in BC_2_ F_3_ population.

**S.No**	**Pyramided lines**	**Screening against five Important pathotypes of bacterial blight pathogen**				
		**DX-002**	**DX-020**	**DX-027**	**DX-148**	**DX-321**
**2B × IRBB 60**						
1	1	3.57 ± 0.30	7.07 ± 1.09	3.83 ± 0.38	4.02 ± 0.32	3.84 ± 0.19
2	2	3.83 ± 0.26	5.95 ± 0.47	4.30 ± 0.26	4.17 ± 0.15	4.20 ± 0.35
3	3	1.33 ± 0.12	5.97 ± 0.46	6.87 ± 1.28	4.03 ± 0.12	4.47 ± 0.20
4	4	3.52 ± 0.29	6.03 ± 0.97	3.72 ± 0.55	4.13 ± 0.41	6.10 ± 0.40
5	5	3.83 ± 0.44	3.72 ± 0.21	3.50 ± 0.29	6.43 ± 0.93	7.03 ± 1.28
6	6	1.15 ± 0.15	3.92 ± 0.12	4.15 ± 0.23	3.33 ± 0.29	7.22 ± 1.14
7	7	4.30 ± 0.21	3.64 ± 0.36	7.53 ± 1.28	4.28 ± 0.20	6.40 ± 0.76
8	8	4.27 ± 0.27	7.57 ± 0.52	4.20 ± 0.21	3.53 ± 0.18	3.60 ± 0.15
9	9	5.43 ± 0.44	4.17 ± 0.35	4.53 ± 0.50	4.17 ± 0.35	3.03 ± 0.50
10	10	3.43 ± 0.26	5.03 ± 0.44	4.12 ± 0.29	6.33 ± 0.64	4.13 ± 0.38
11	11	3.73 ± 0.33	4.03 ± 0.50	4.17 ± 0.35	7.22 ± 0.94	7.32 ± 1.09
12	12	4.03 ± 0.38	7.03 ± 0.45	6.03 ± 0.88	3.67 ± 0.12	13.77 ± 0.69
**23 B × IRBB 60**						
13	1	5.57 ± 0.17	7.93 ± 0.97	4.20 ± 0.60	7.77 ± 0.72	8.77 ± 0.47
14	2	3.70 ± 0.21	6.83 ± 0.18	3.73 ± 0.33	4.0 ± 70.38	7.37 ± 0.50
15	3	6.80 ± 0.49	0.93 ± 0.35	4.10 ± 0.36	4.1 ± 00.23	4.00 ± 0.42
16	4	8.10 ± 2.83	4.20 ± 0.06	5.07 ± 0.38	7.60 ± 0.47	7.33 ± 2.36
17	5	3.23 ± 0.32	3.43 ± 0.30	3.70 ± 0.31	5.92 ± 0.91	3.57 ± 0.32
18	6	3.55 ± 0.32	3.23 ± 0.32	0.98 ± 0.32	1.57 ± 0.22	7.40 ± 0.76
19	7	3.17 ± 0.12	3.43 ± 0.26	3.60 ± 0.29	3.50 ± 0.35	1.00 ± 0.31
20	8	3.83 ± 0.18	3.70 ± 0.15	3.57 ± 0.52	3.53 ± 0.30	3.83 ± 0.18
21	9	3.21 ± 0.26	3.39 ± 0.36	3.32 ± 0.20	3.45 ± 0.24	1.42 ± 0.31
**24 B × IRBB 60**						
22	1	3.66 ± 0.32	3.73 ± 0.33	6.97 ± 0.97	3.70 ± 0.31	3.57 ± 0.32
23	2	3.43 ± 0.32	0.97 ± 0.35	3.63 ± 0.32	0.57 ± 0.20	7.40 ± 0.76
24	3	3.27 ± 0.11	3.48 ± 0.26	3.20 ± 0.28	3.59 ± 0.39	1.10 ± 0.31
25	4	3.43 ± 0.18	3.70 ± 0.15	3.57 ± 0.52	3.53 ± 0.30	3.83 ± 0.18
26	5	2.04 ± 0.22	1.55 ± 0.28	0.68 ± 0.30	2.67 ± 0.28	2.98 ± 0.15
27	6	3.25 ± 0.32	3.29 ± 0.31	0.98 ± 0.42	1.97 ± 0.22	5.30 ± 0.76
28	7	2.04 ± 0.22	1.65 ± 0.28	0.68 ± 0.30	2.67 ± 0.28	2.98 ± 0.15
29	8	2.84 ± 0.32	2.57 ± 0.26	1.64 ± 0.35	2.36 ± 0.25	2.55 ± 0.25
30	9	2.38 ± 0.25	1.55 ± 0.28	1.58 ± 0.33	2.60 ± 0.65	2.73 ± 0.18
31	2B (Recurrent parent)	24.47 ± 1.09	26.53 ± 1.21	26.27 ± 2.59	26.20 ± 2.43	9.30 ± 0.15
32	23B (Recurrent parent)	26.27 ± 1.85	27.03 ± 1.91	26.23 ± 2.46	13.07 ± 0.38	27.10 ± 2.26
33	24B Recurrent parent)	28.50 ± 1.06	16.30 ± 0.47	12.47 ± 0.84	13.43 ± 0.60	27.13 ± 1.65
34	IR24 (Susceptible check)	23.89 ± 2.49	21.71 ± 1.39	19.40 ± 2.09	23.16 ± 3.01	26.54 ± 2.55
35	IRBB60 (Donor parent)	4.33 ± 0.29	4.43 ± 0.92	3.30 ± 0.17	0.83 ± 0.23	4.53 ± 0.92

*The values are given in cm*.

### Hybrid development

Presence/absence of both fertility restorer genes, *Rf3* and *Rf4* were verified in 30 homozygous. Two plants each in CO 2B × IRBB60 and CO 24B × IRBB60 combination, one plant in CO 23B x IRBB60 shows negative for both *Rf3* and *Rf4* fertility-restorer genes. Based on the agronomic traits, a single plant from CO 2B, CO 23B, and CO 24B cross combinations was selected and test crossed with respective CMS lines. The test crossed plants are intact for all three resistance genes and completely sterile for both pollen and spikelet fertility. Two more back crosses are needed to convert these lines into a male sterile source. Restorer lines, AD 08009 AD 09525, IET 19863, 20885, 20897 20898 of WA cytoplasmic source were crossed with improved lines of CO 2B, CO 23B, and CO 24B and hybrids were tested for their superiority for yield. Of several hybrid combinations, the hybrids combinations *viz.*, CO 23A × AD08009R and CO 24A × IET20898R were found to be stable over locations with high yield (Table S1).

## Discussion

To overcome agrochemical pollution and time consuming conventional breeding methods, we should develop long lasting resistance varieties in the management of BB disease (Khush et al., 1989; Gnanamanickam et al., 1999). With the current advances in DNA marker based breeding and gene pyramiding, it becomes more comfortable in developing BB resistant rice varieties without compromising their yield and grain quality. Tagging of pyramided resistance genes and identifying the presence of multiple genes showing resistance to different races of *Xoo* pathogen through functional markers would provide a valid and reproducible selection strategy. Functional marker assisted selection played a key role in improving cytoplasmic male sterile lines by incorporating the three bacterial blight resistance genes (*Xa21, xa13*, and *xa5)* through back-crossing and selfing. These genes conferred a tough and wide spectrum resistance to *Xoo* starins distributed worldwide where rice is grown.

More than 38 major genes, named from *Xa1* to *Xa38* (Bhasin et al., 2011) have been identified from various environments and have conferred resistance to different strains of *Xoo* pathogens. The rice gene *Xa21* has got its own significance in breeding practices for developing rice varieties with bacterial blight resistance due to its wide range of resistance against bacterial blight disease (Huang et al., 1997; Li et al., 2001; Pradhan et al., 2015; Luo et al., 2016).

*xa5* is an important BB resistance gene because of its recessive nature and it does not belong to the emblematic structural classes of resistance genes. *xa5* gene, which depends on the effector genes present in the pathogens, is broadly effective against the Xoo races(Jiang et al., 2006). The efficiency of *xa5* in combating *Xanthomonas oryzae pv. Oryzae* depends mainly on TAL (Transcription Activation-like) effector genes, but regrettably futile in combating disease by strains in which S gene is expressed. The *xa5* gene, though provides vertical resistance, but not associated with dominant resistance genes or clusters of resistance gene analogs (Ilag et al., 2000; Wang et al., 2001). The race specific *xa13* mediated resistance is exclusively resistance to the most virulent Philippines *Xoo* race 6 which is mostly not conquered by other reported R genes. The fully recessive *xa13* is insensitive to TAL effector PthXo1 and therefore highly resistant to the PthXo1 dependent virulence of pathogens (Khush and Angeles, 1999). Improved resistance was achieved by the suppression of either dominant or recessive allele, but also resulted in male sterility pointing that this gene plays a role in pollen development as well as in bacterial growth dependent modular (Chu et al., 2006).

The combination of these three genes *(Xa21*+ *xa5* +*xa13*) can accomplish a tough and broad spectrum resistance in many BB prone rice growing areas in India. The order of gene combinations in conferring resistance *xa5* + *xa13* < *xa5* + *Xa21* < *xa13* + *Xa21* < *xa5* + *xa13* + *Xa21* indicated the degree of severity of disease. Lines with *Xa21* in combination with either *xa5, xa13* or both have shown promise advocating the utility of *Xa21* in achieving higher levels of resistance in rice as reported earlier (Sanchez et al., 2000; Singh et al., 2001). The present work was thus focused not only to pyramid both recessive and dominant genes for resistance, but to keep the maintaining ability of parental lines for sterility.

The genes of BB resistance should be incorporated into both the parental lines of the hybrid for the expression of desired level of resistance. To incorporate resistance genes into CMS lines, first, the genes must be transferred to the maintainer's background. Thirty homozygous lines with high resistance to bacterial blight in the background of CO 2A, CO 23 A, and CO 25 A were developed. They were again screened for the presence of fertility restorer genes, *Rf 3* and *Rf 4* through molecular marker linked to these genes. SSR markers DRCG-*RF4*-14/DRCG-*RF4*-8/RM6100 for the *Rf4* locus ad DRRM-RF3-5/DRRM-RF3-10 for *Rf3* linked to fertility restoration genes (*Rf3, Rf4*) were employed for detecting efficacious non-fertility restorer plants for WA-CMS system.

This helped us to identify the plants possessing resistance gene, which also had non restoring alleles in homozygous condition at BC_2_F_3_ generation. Sundaram et al. (2007) successfully pyramided the resistance genes (*Xa21*+ *xa5* +*xa13*) into Samba Mashuri, an elite rice variety having high yield grain and cooking quality. Using rice microsatellite (RM) or simple sequence repeat (SSR) markers, *Rf3* and *Rf4* loci have been identified in different donors (He et al., 2002; Sattari et al., 2006; Bazrkar et al., 2008; Nematzadeh and Kiani, 2010). Marker combination of DRCG-*RF4*-14/DRCG-*RF4*-8/RM6100 for the *Rf4* locus and DRRM-RF3-5/DRRM-RF3-10 for *Rf3* showed the maximum efficiency to select the non-fertility restorer genes. The earlier attempts for MAS have used STS markers (Nas et al., 2003; Sattari et al., 2007) and either *Rf3* or *Rf4* markers (Nas et al., 2003; Sheeba et al., 2009; Ngangkham et al., 2010; Pranathi et al., 2016; Katara et al., 2017), only one study used two STS markers from *Rf3* and *Rf4* loci with selection accuracy of 100% in 13 R lines (Sattari et al., 2007). Selection accuracy of 94.9% in a set of 21 restorer lines with RM6100 from *Rf4* was reported by Sheeba et al., (2009). Sattari et al., (2007) and Bazrkar et al., (2008) used the sequence tagged sites (STS) RG140/PvuII and S10019/BstUI in MAS for fertility restoration genes *Rf3* on chromosome 1 and *Rf4* on chromosome 10 in rice. This suggests that two dominant genes *Rf3* and *Rf4* seem to control the fertility restoration based on above studies. The effect of one of the two dominant genes (*Rf3*) in restoring fertility appears to be strong and as good as the two together (*Rf3Rf4*) while the other gene (*Rf4*) showed weak restoration in rice (Mahalingam and Saraswathi, 2016). These results show two dominant genes *Rf3* and *Rf4* to control the fertility restoration. If *Rf3, Rf4* genes are present together, the effect of one of the two genes in restoring fertility appeared to be stronger than the other. This indicated that the recessive gene alone control the fertility restoration (Chu et al., 2006). From the above work, it was observed that in the presence of *Rf3 and Rf4* genes plants shows complete fertility restoration. With the use of *Rf3* (DRRM-RF3-5/DRRM-RF3-10) and *Rf4* (DRCG-*RF4*-14/DRCG-*RF4*-8/RM6100) markers, the positive plants were ignored and the plants which are negative for the *Rf3, Rf4* genes were selected which would never restore fertility and shows only sterile, also phenotypically similar to the recurrent parent. The above selected negative plants were confirmed as maintainer by crossing the improved lines with corresponding male sterile lines (CO 2A, CO 23A, and CO 24A).

A single plant in each cross was identified with all BB resistance and without fertility restorer genes- *Rf3* and *Rf4*. These plants are highly resistance to all five races of *Xoo* pathogen and maintain 100% male sterility in the test crosses. Non-recovery of recessive resistance gene xa5 with other genes was observed in earlier studies of Dokku et al. (2013). But in this study no such instance, was observed. Using the improved version of parental lines 2B, 23 B, and 24 B, number of hybrids were developed. Two hybrids, CO 23B × AD08009R and CO 24B × IET20898R, were found to be stable over locations with high yield. This new hybrid with resistance against bacterial blight will increase the rice productivity. Using functional markers, durable resistance cytoplasmic male sterile lines were developed in this study through MAS. It demonstrates the successful transfer of recessive and dominant genes with the intact recurrent genome.

## Conclusion

We have introgressed three BB resistance genes (*xa5, xa13*, and *Xa21*) which would be desirable to achieve durable and broad spectrum resistance in the maintainer lines of hybrid rice through functional markers assisted backcross breeding. Thirty homozygous plants, 12 in CO 2B × IRBB60, nine each in CO 23B × IRBB60 and CO 24B × IRBB60 were identified and finally single line in each male sterile background without out fertility restorer genes, *Rf3* and *Rf4* was developed. The improved versions of male sterile and maintainer lines of stable CMS lines CO 2A, CO 23A, and CO 24A can form the base to develop new wide adoptable heterotic hybrids with resistance against the most destructive diseases on rice. Using these lines, two high yielding and stable hybrid combinations- CO 23B × AD08009R and CO 24B × IET20898R were identified in the current study.

## Author contributions

JR conceived and designed the experiment. PS has performed the experiments. GA involved part of the experiment and helped in revising the manuscript. JR and RS involved in the development of CMS lines. RC and all authors prepared and approved the final version of the manuscript.

### Conflict of interest statement

The authors declare that the research was conducted in the absence of any commercial or financial relationships that could be construed as a potential conflict of interest.

## Supplementary material

Datasheet S1 PCR details for the introgression of xa5, xa13 and Xa21 and *Rf3* and *Rf4* genes.

Table S1 Comparative hybrid yield performance from the cross of BB improved CMS line and identified BB improved R line.

## Abbreviations

WA: wild abortive
CMS: cytoplasmic male sterility
*Rf*: fertility restorer
SSR: Simple sequence repeats
*Xoo*: *Xanthomonas oryzae pv. oryzae*
BB: Bacterial blight
MAS: Marker assisted selection
DAS: Date after sowing.

## References

[B1] Andersen J. R. Lübberstedt T. (2003). Functional markers in plants. Trends Plant Sci. 8, 554–560. 10.1016/j.tplants.2003.09.010 14607101

[B2] Balachiranjeevi C. Bhaskar N. S. Abhilash V. Akanksha S. Viraktamath B. C. Madhav M. S. (2015). Marker-assisted introgression of bacterial blight and blast resistance into DRR17B, an elite, fine-grain type maintainer line of rice. Mol. Breed. 35:151 10.1007/s11032-015-0348-8

[B3] Basavaraj S. H. Singh V. K. Singh A. Singh A. Singh A. Anand D. (2010). Marker-assisted improvement of bacterial blight resistance in parental lines of Pusa RH10, a superfine grain aromatic rice hybrid. Mol. Breed. 26, 293–305. 10.1007/s11032-010-9407-3

[B4] Bazrkar L. Ali A. J. Babaeian N. A. Ebadi A. A. Allahgholipour M. Kazemitabar K. (2008). Tagging of four fertility restorer loci for wild abortive—cytoplasmic male sterility system in rice (*Oryza sativa* L.) using microsatellite markers. Euphytica 164, 669–677. 10.1007/s10681-008-9667-8

[B5] Bhasin H. Bhatia D. Raghuvanshi S. Lore J. S. Sahi G. K. Kaur B. (2011). New PCR-based sequence-tagged site marker for bacterial blight resistance gene Xa38 of rice. Mol. Breed. 30, 607–611. 10.1007/s11032-011-9646-y

[B6] Chen D.-H. Ronald P. (1999). A rapid DNA minipreparation method suitable for AFLP and other PCR applications. Plant Mol. Biol. Report. 17, 53–57. 10.1023/a:1007585532036

[B7] Chen S. Lin X. Xu C. Zhang Q. (2000). improvement of bacterial blight resistance of ‘Minghui 63’, an elite restorer line of hybrid rice, by molecular marker-assisted selection. Crop Sci. 40:239 10.2135/cropsci2000.401239x

[B8] Chu Z. Yuan M. Yao J. Ge X. Yuan B. Xu C.. (2006). Promoter mutations of an essential gene for pollen development result in disease resistance in rice. Genes Dev. 20, 1250–1255. 10.1101/gad.1416306 16648463 PMC1472899

[B9] Dokku P. Das K. M. Rao G. J. N. (2013). Pyramiding of four resistance genes of bacterial blight in Tapaswini, an elite rice cultivar, through marker-assisted selection. Euphytica 192, 87–96. 10.1007/s10681-013-0878-2

[B10] Gnanamanickam S. S. Priyadarisini V. B. Narayanan N. N. Vasudevan P. Kavitha S. (1999). An overview of bacterial blight disease of rice and strategies for its management. Curr. Sci. 77, 1435–1444.

[B11] He G. H. Wang W. M. Liu G. Q. Hou L. Xiao Y. H. Tang M.. (2002). Mapping of two fertility restoring genes for WA cytoplasmic male sterility in Minghui63 using SSR markers. Acta Genet. Sin. 29, 798–802. 12561227

[B12] Huang B. Xu J. Y. Hou M. S. Ali J. Mou T. M. (2012). Introgression of bacterial blight resistance genes Xa7, Xa21, Xa22 and Xa23 into hybrid rice restorer lines by molecular marker-assisted selection. Euphytica 187, 449–459. 10.1007/s10681-012-0758-1

[B13] Huang N. Angeles E. R. Domingo J. Magpantay G. Singh S. Zhang G. (1997). Pyramiding of bacterial blight resistance genes in rice: marker-assisted selection using RFLP and PCR. Theor. Appl. Genet. 95, 313–320. 10.1007/s001220050565

[B14] Ilag L. L. Yadav R. C. Huang N. Ronald P. C. Ausubel F. M. (2000). Isolation and characterization of disease resistance gene homologues from rice cultivar IR64. Gene 255, 245–255. 10.1016/s0378-1119(00)00333-4 11024284

[B15] Ingvardsen C. R. Schejbel B. Lübberstedt T. (2008). Functional markers in resistance breeding. Progr. Bot. 69, 61–87. 10.1007/978-3-540-72954-9_3

[B16] Iyer-Pascuzzi A. S. Mccouch S. R. (2007). Functional markers for xa5-mediated resistance in rice (*Oryza sativa*, L.). Mol. Breed. 19, 291–296. 10.1007/s11032-006-9055-9

[B17] Jiang G.-H. Xia Z.-H. Zhou Y.-L. Wan J. Li D.-Y. Chen R.-S.. (2006). Testifying the rice bacterial blight resistance gene xa5 by genetic complementation and further analyzing xa5 (Xa5) in comparison with its homolog TFIIAγ1. Mol. Genet. Genomics 275, 354–366. 10.1007/s00438-005-0091-7 16614777

[B18] Katara J. L. Verma R. L. Nayak D. Ngangkham U. Ray S. Subudhi H. (2017). Frequency and fertility restoration efficiency of Rf3 and Rf4 genes in Indian rice. Plant Breed. 136, 74–82. 10.1111/pbr.12401

[B19] Khush G. S. Angeles E. R. (1999). A new gene for resistance to race 6 of bacterial blight in rice, *Oryza sativa* L. Rice Genet Newsl. 16, 92–93.

[B20] Khush G. S. Mackil D. J. Sidhu G. S. (1989). Breeding rice for resistance to bacterial blight, in Bacterial Blight of Rice, ed Ri I. R. (Manila: IRRI), 207–217.

[B21] Kumar V. A. Balachiranjeevi C. H. Naik S. B. Rambabu R. Rekha G. Harika G.. (2016). Marker-assisted improvement of the elite restorer line of rice, RPHR-1005 for resistance against bacterial blight and blast diseases. J. Genet. 95, 895–903. 10.1007/s12041-016-0711-5 27994188

[B22] Li Z.-K. Sanchez A. Angeles E. Singh S. Domingo J. Huang N.. (2001). Are dominant genes similar?: a case study of rice R genes and *Xanthomonas oryzae* pv *oryzae* races. Genetics 159, 757–765. 11606550 10.1093/genetics/159.2.757 PMC1461810

[B23] Luo Y. Ma T. Zhang A. Ong K. H. Li Z. Yang J.. (2016). Marker-assisted breeding of the rice restorer line Wanhui 6725 for disease resistance, submergence tolerance and aromatic fragrance. Rice 9:66. 10.1186/s12284-016-0139-9 27905090 PMC5130935

[B24] Mahalingam A. Saraswathi R. (2016). Genetics of fertility restoration of Wild Abortive system based Cytoplasmic Male Sterility (CMS) in Hybrid rice (*Oryza sativa* L.). J. Inno. Agri. 3, 1–9.

[B25] Nas T. M. S. Casal C. L. Li Z. Virmani S. S. (2003). Application of molecular markers for identification of restorers. Rice Genet. Newsl. 20, 69–71.

[B26] Nematzadeh G. H. Kiani G. (2010). Genetic analysis of fertility restoration genes for WA type cytoplasmic male sterility in Iranian restorer rice line DN-33-18. Afr. J. Biotechnol. 9, 6273–6277.

[B27] Ngangkham U. Parida S. K. De S. Kumar K. A. R. Singh A. K. Singh N. K. (2010). Genic markers for wild abortive (WA) cytoplasm based male sterility and its fertility restoration in rice. Mol. Breed. 26, 275–292. 10.1007/s11032-010-9397-1

[B28] Perumalsamy S. Bharani M. Sudha M. Nagarajan P. Arul L. Saraswathi R. (2010). Functional marker-assisted selection for bacterial leaf blight resistance genes in rice (*Oryza sativa* L.). Plant Breed. 129, 400–406. 10.1111/j.1439-0523.2009.01705.x

[B29] Pradhan S. K. Nayak D. K. Mohanty S. Behera L. Barik S. R. Pandit E.. (2015). Pyramiding of three bacterial blight resistance genes for broad-spectrum resistance in deepwater rice variety, Jalmagna. Rice 8:19. 10.1186/s12284-015-0051-8 26054243 PMC4489969

[B30] Pranathi K. Viraktamath B. C. Neeraja C. N. Balachandran S. M. Prasad A. S. H. Rao P. K. (2016). Development and validation of candidate gene-specific markers for the major fertility restorer genes, Rf4 and Rf3 in rice. Mol. Breed. 36:145 10.1007/s11032-016-0566-8

[B31] Sanchez A. Brar D. Huang N. Li Z. Khush G. (2000). Sequence tagged site marker-assisted selection for three bacterial blight resistance genes in rice. Crop Sci. 40:792 10.2135/cropsci2000.403792x

[B32] Sattari M. Kathiresan A. Gregorio G. B. Hernandez J. E. Nas T. M. Virmani S. S. (2006). Development and use of a two-gene marker-aided selection system for fertility restorer genes in rice. Euphytica 153, 35–42. 10.1007/s10681-006-9213-5

[B33] Sattari M. Kathiresan A. Gregorio G. B. Virmani S. S. (2007). Comparative genetic analysis and molecular mapping of fertility restoration genes for WA, Dissi, and Gambiaca cytoplasmic male sterility systems in rice. Euphytica 160, 305–315. 10.1007/s10681-007-9498-z

[B34] Shanti M. L. Devi G. L. Kumar G. N. Shashidhar H. (2010). Molecular marker-assisted selection: a tool for insulating parental lines of hybrid rice against bacterial leaf blight. Int. J. Plant Pathol. 1, 114–123. 10.3923/ijpp.2010.114.123

[B35] Sheeba N. K. Viraktamath B. C. Sivaramakrishnan S. Gangashetti M. G. Khera P. Sundaram R. M. (2009). Validation of molecular markers linked to fertility restorer gene(s) for WA-CMS lines of rice. Euphytica 167, 217–227. 10.1007/s10681-008-9865-4

[B36] Singh S. Sidhu J. S. Huang N. Vikal Y. Li Z. Brar D. S. (2001). Pyramiding three bacterial blight resistance genes (xa5, xa13 and Xa21) using marker-assisted selection into indica rice cultivar PR106. TAG Theor. Appl. Genet. 102, 1011–1015. 10.1007/s001220000495

[B37] Sundaram R. M. Vishnupriya M. R. Biradar S. K. Laha G. S. Reddy G. A. Rani N. S. (2007). Marker assisted introgression of bacterial blight resistance in Samba Mahsuri, an elite indica rice variety. Euphytica 160, 411–422. 10.1007/s10681-007-9564-6

[B38] Suresh P. B. Srikanth B. Kishore V. H. Rao I. S. Vemireddy L. R. Dharika N. (2012). Fine mapping of Rf3 and Rf4 fertility restorer loci of WA-CMS of rice (*Oryza sativa* L.) and validation of the developed marker system for identification of restorer lines. Euphytica 187, 421–435. 10.1007/s10681-012-0737-6

[B39] Varshney R. K. Graner A. Sorrells M. E. (2005). Genomics-assisted breeding for crop improvement. Trends Plant Sci. 10, 621–630. 10.1016/j.tplants.2005.10.004 16290213

[B40] Wang Z. Taramino G. Yang D. Liiu G. Tingey S. V. Miano G. H.. (2001). Rice ESTs with disease-resistance gene-or-defense-response gene-like sequences mapped to regions containing major resistance genes or QTLs. Mol. Genet. Genomics 265, 302–310. 10.1007/s004380000415 11361341

[B41] Zhang Q. (2009). Genetics and Improvement of Bacterial Blight Resistance of Hybrid Rice in China. Rice Sci. 16, 83–92. 10.1016/s1672-6308(08)60062-1

[B42] Zhang Q. Wang C. L. Zhao K. J. Yang W. C. Qiao F. Zhou Y. L. (2002). Development of near-isogenic line CBB23 with a new resistance gene to bacterial blight in rice and its application. Chin. J. Rice Sci. 16, 206–210.

[B43] Zhou Y.-L. Uzokwe V. N. Zhang C.-H. Cheng L.-R. Wang L. Chen K. (2011). Improvement of bacterial blight resistance of hybrid rice in China using the Xa23 gene derived from wild rice (*Oryza rufipogon*). Crop Protect. 30, 637–644. 10.1016/j.cropro.2010.12.002

