# Supplementary material for: Functional Marker Assisted Improvement of Stable Cytoplasmic Male Sterile Lines of Rice for Bacterial Blight Resistance
Source: Front Plant Sci. 2017 Jun 29;8:1131. doi: 10.3389/fpls.2017.01131 (PMC5489691; doi:10.3389/fpls.2017.01131)
Supplement: Datasheet S1 — PCR details for the introgression of xa5, xa13 and Xa21 and Rf3 and Rf4 genes. [file DataSheet1.PDF]

xa5

94^0 – 4 min
94^0 – 1 min }
56^0 – 1 min } 35 cycles
72^0 – 1 min }
72^0 – 7 min

xa13

94^0 – 5 min
94^0 – 1 min }
59^0 – 1 min } 35 cycles
72^0 – 1 min 30s }
72^0 – 7 min

Xa21

94^0 – 5 min
94^0 – 45s }
65^0 – 1 min } 35 cycles
72^0 – 1 min 30s }
72^0 – 7 min

DRCG-Rf 4-14

94^0 – 5 min
94^0 – 1 min }
53.4^0 – 1 min } 35 cycles
72^0 – 1 min 30s }
72^0 – 7 min

DRRM-Rf 3-5

94^0 – 5 min
94^0 – 1 min }
60.5^0 – 1 min } 35 cycles
72^0 – 1 min 30s }
72^0 – 7 min

DRCG-Rf 4-8

94^0 – 5 min
94^0 – 1 min }
48.5^0 – 1 min } 35 cycles
72^0 – 1 min 30s }
72^0 – 7 min

DRRM-Rf 3-10

94^0 – 5 min
94^0 – 1 min }
54.5^0 – 1 min } 35 cycles
72^0 – 1 min 30s }
72^0 – 7 min
