# Supplementary material for: Functional Marker Assisted Improvement of Stable Cytoplasmic Male Sterile Lines of Rice for Bacterial Blight Resistance
Source: Front Plant Sci. 2017 Jun 29;8:1131. doi: 10.3389/fpls.2017.01131 (PMC5489691; doi:10.3389/fpls.2017.01131)
Supplement: Table S1 — Comparative hybrid yield performance from the cross of BB improved CMS line and identified BB improved R line. [file Table1.PDF]

Table S1. Mean performance of CMS lines with 21 restorers in rice for different quantitative traits

Sl . No	Hybrid combinations	DTF	PH	NPT	FL	FB	PL	PB	SB	NFG	TNSP	PF	SF	HGW	SPY
1	**COMS 23A × IET 19863 R**	88.50	96.27	17.98**	40.00	1.50	28.18	12.44	43.51	172.83**	191.57**	96.23**	90.21**	2.00**	54.45**
2	**COMS 23A × IET 20881 R**	103.00	94.99	16.49	37.25	**1.85****	26.01	15.37**	68.17**	213.50**	226.65**	99.24**	94.19**	1.54	46.06**
3	**COMS 23A × IET 20885 R**	96.50	104.00**	17.50**	37.50	1.80**	31.23**	15.25**	66.51**	211.11**	232.77**	95.31**	90.69**	1.75**	55.32**
4	**COMS 23A × IET 20897 R**	90.50	94.50	17.82**	49.20**	1.60**	29.25	15.68**	63.89**	207.00**	224.33**	96.33**	92.27**	1.75**	50.81**
5	**COMS 23A × IET 20898 R**	89.50	106.16**	17.76**	40.00	1.50	28.25	15.28**	62.30**	194.39**	203.42**	99.17**	**95.56****	1.32	37.80**
6	**COMS 23A × IET 20937 R**	91.50	92.00	17.68**	44.75**	1.40	28.07	14.16**	55.63**	167.20**	180.68**	95.98**	92.54**	1.62	38.31**
7	**COMS 23A × IET 20945 R**	99.50	103.50	13.95	48.75**	1.45	28.86	13.66	52.22	115.11	142.77	84.47	80.63	1.67	21.51
8	**COMS 23A × AD 06084 R**	108.50**	100.50	11.50	36.40	1.40	31.41**	11.50	51.00	90.67	112.34	82.67	80.72	1.46	13.84
9	**COMS 23A × AD 07073 R**	98.00	89.00	16.42	35.25	1.50	27.49	15.32**	48.81	152.69**	168.50	91.14	90.63**	1.72**	36.97**
10	**COMS 23A × AD 07083 R**	97.50	103.42	15.50	33.75	1.45	26.61	10.63	43.83	146.36	161.67	90.03	90.70**	1.55	32.68
11	**COMS 23A × AD 08005 R**	88.00	93.50	12.75	35.75	1.40	26.25	13.00	50.50	106.50	129.01	85.11	82.55	1.87**	20.33
12	**COMS 23A × AD 08009 R**	93.50	97.00	17.68**	36.40	1.50	27.25	14.61**	57.47**	167.39**	180.50**	98.12**	92.75**	1.74**	38.18**
13	**COMS 23A × AD 09194 R**	90.00	95.50	17.63**	31.80	1.30	28.25	15.65**	58.82**	149.06**	162.00	96.41**	92.01**	1.45	32.09
14	**COMS 23A × AD 09216 R**	100.00	88.50	16.82	**27.90**	1.20	26.84	13.78	55.75	156.50**	171.61**	96.51**	91.20**	1.46	32.00
15	**COMS 23A × AD 09223 R**	93.00	94.50	16.00	32.62	1.45	26.25	13.97	47.08	160.50**	175.00**	96.46**	91.43	1.66	36.09**
16	**COMS 23A × AD 09522R**	101.00	96.50	16.61	40.25	1.35	27.75	12.50	57.41**	168.50**	184.60**	95.83**	91.28**	1.77**	41.73**
17	**COMS 23A × AD 09523R**	103.00	106.28**	14.50	41.00**	1.50	27.25	13.00	52.90	97.91	120.67	83.99	81.13	1.41	15.57
18	**COMS 23A × AD 09525R**	110.50**	106.25**	13.78	39.25	1.52	26.25	11.50	51.21	111.76	136.85	83.64	81.66	1.55	20.51
19	**COMS 23A × AD 09530R**	118.00**	107.00**	15.98	35.90	1.30	28.78	13.74	52.77	145.67	176.68**	99.33**	82.45	1.66	32.24
20	**COMS 23A × AD 09531R**	109.00**	98.50	17.93**	38.50	1.80**	29.25	15.45**	48.49	189.03**	207.38**	93.03**	91.16**	1.52	40.67**
21	**COMS 23A × AD 09533R**	109.00**	106.00**	14.26	38.25	1.50	25.00	12.50	41.17	147.50	166.50	**100.00****	88.59**	**1.18**	21.49

Contd..

Note: days to 50 percent flowering (DTF, days); plant height (PH, cm); number of productive tillers per plant (NPT, no); flag leaf length (FL, cm); flag leaf breath (FB, cm); panicle length (PL, cm); panicle breath (PB, cm); shoot biomass (SB, g/plant); number of filled grains per panicle (NFG, no); total number of spikelet per panicle (TNSP, no); panicle fertility (PF, no); spikelet fertility (SF, no); hundred grain weight (HGW, g); single plant yield (SPY, g).

Table S1. Contd…

**Sl . No**	**Hybrid combinations**	**DTF**	**PH**	**NPT**	**FL**	**FB**	**PL**	**PB**	**SB**	**NFG**	**TNSP**	**PF**	**SF**	**HGW**	**SPY**
22	**COMS 24A × IET 19863 R**	91.50	95.99	23.09**	37.00	1.55	28.00	10.12	**29.61**	143.83	151.17	95.58**	95.16**	**2.13****	59.14**
23	**COMS 24A × IET 20881 R**	108.50**	104.50**	12.50	37.00	1.50	30.75**	12.50	62.00**	177.78**	217.28**	96.05**	81.82	1.93**	31.69
24	**COMS 24A × IET 20885 R**	111.00**	121.25**	11.75	39.25	1.50	27.13	14.25**	62.75**	113.28	136.50	85.00	82.99	1.30	13.79
25	**COMS 24A × IET 20897 R**	93.00	106.50**	21.75**	47.17**	1.80**	31.00**	15.73**	59.97**	229.66**	245.00**	99.65**	93.74**	1.77**	70.15**
26	**COMS 24A × IET 20898 R**	93.00	106.61**	16.49	42.25**	1.80**	30.00**	14.50**	63.26**	201.32**	216.67**	98.21**	92.92**	1.95**	50.57*
27	**COMS 24A × IET 20937 R**	108.00**	94.75	19.50**	49.00**	1.60**	26.75	11.50	49.17	96.98	117.81	85.51	82.32	1.66	24.10
28	**COMS 24A × IET 20945 R**	97.50	106.25**	18.91**	45.25**	1.41	**31.59****	15.59**	68.50**	234.75**	257.05**	91.95	91.33**	1.93**	62.06**
29	**COMS 24A × AD 06084 R**	99.50	114.00**	15.28	32.25	1.30	31.25**	12.16	51.66	109.00	134.50	82.21	81.04	1.32	19.13
30	**COMS 24A × AD 07073 R**	99.00	92.33	14.66	38.00	1.40	25.34	14.00	45.66	90.50	112.36	82.41	80.54	1.79**	18.95
31	**COMS 24A × AD 07083 R**	95.00	105.50**	16.00	30.00	1.20	30.50**	12.00	42.44	140.83	150.00	90.08	93.90**	1.56	34.76
32	**COMS 24A × AD 08005 R**	85.50	96.00	17.61**	37.30	1.30	27.75	**16.30****	50.00	179.48**	198.27**	96.64**	90.53**	1.75**	44.73**
33	**COMS 24A × AD 08009 R**	103.50	100.08	11.50	32.83	1.30	29.19	12.84	47.00	105.25	126.45	85.85	83.24	1.73**	16.37
34	**COMS 24A × AD 09194 R**	97.00	96.25	13.25	36.75	1.45	28.88	10.50	46.17	109.00	134.50	84.09	81.04	1.35	15.38
35	**COMS 24A × AD 09216 R**	98.50	111.00**	11.50	36.25	1.30	28.75	13.00	50.77	95.50	116.00	85.03	82.33	1.44	**13.74**
36	**COMS 24A × AD 09223 R**	94.00	88.00	16.00	33.30	1.50	27.25	12.84	47.33	163.50**	180.03**	91.72	90.06**	1.62	37.73**
37	**COMS 24A × AD 09522R**	87.00	95.50	17.18**	39.50	1.35	28.75	14.50**	64.44**	187.00**	203.89**	92.77**	91.72**	1.72**	45.35**
38	**COMS 24A × AD 09523R**	107.00**	108.50**	19.68**	47.25**	1.70**	28.75	15.17**	**72.00****	187.00**	207.60**	99.33**	90.08**	1.47	47.92**
39	**COMS 24A × AD 09525R**	114.50**	109.55**	22.77**	44.25**	1.50	27.00	15.00**	61.17**	231.00**	254.71**	98.92**	90.69**	1.52	**73.60****
40	**COMS 24A × AD 09530R**	102.00	101.00	18.41**	39.25	1.25	28.13	13.50	57.00**	141.32	175.34**	98.03**	80.60	1.63	35.49**
41	**COMS 24A × AD 09531R**	107.00**	99.00	11.50	33.25	1.30	26.50	11.50	42.83	94.71	117.56	84.30	80.57	1.46	14.34
42	**COMS 24A × AD 09533R**	109.50**	115.25**	11.50	40.40	1.20	29.75**	12.00	45.28	99.67	123.24	84.88	80.89	1.55	16.06

Note: days to 50 percent flowering (DTF, days); plant height (PH, cm); number of productive tillers per plant (NPT, no); flag leaf length (FL, cm); flag leaf breath (FB, cm); panicle length (PL, cm); panicle breath (PB, cm); shoot biomass (SB, g/plant); number of filled grains per panicle (NFG, no); total number of spikelet per panicle (TNSP, no); panicle fertility (PF, no); spikelet fertility (SF, no); hundred grain weight (HGW, g); single plant yield (SPY, g).
